# Oral Health Status and Associated Factors in a Stratified Cluster Sample of Marginalized Palestinian Schools: A Cross-sectional Study

**DOI:** 10.3290/j.ohpd.b1694115

**Published:** 2021-07-15

**Authors:** Elham Kateeb, Motasem Hamdan, Julian Fisher

**Affiliations:** a Associate Professor, Department of Periodontology and Preventive Dentistry, Al-Quds University, Jerusalem, State of Palestine; Public Policy Center, the University of Iowa, Iowa City, IA, USA. Study design, sampling, developed the study instrument, directly supervised the data collection process, analysed the data, wrote the manuscript, read and approved the final manuscript.; b Professor, College of Public Health, Al-Quds University, Al-Quds University, Jerusalem, State of Palestine. Study concept and design planning, read and approved the manuscript.; c Research Assistant Professor, Department of Public Health & Preventive Medicine, State University of New York Upstate Medical University, Syracuse, NY, USA. Concept development and manuscript editing, read and approved the manuscript.

**Keywords:** adolescents, body mass index, dental caries, dietary habits, DMFT

## Abstract

**Purpose:**

To assess factors related to the prevalence of dental caries among adolescent schoolchildren attending marginalised schools in the West Bank area of Palestine.

**Materials and Methods:**

A cross-sectional study was conducted in schools participating in the School Support Program (SSP). Fifty schools identified as marginalised by the SSP were stratified by district, student gender and grade level to select a random sample of 20 schools. Students in the 6th and 9th grades were screened by senior dental students to collect data about their weight, height, gingival health and caries experience. In addition, a structured in-person questionnaire was used to collect data about students’ oral hygiene practices, dietary habits, mother’s education and father’s employment.

**Results:**

In total, 1282 students completed interviews and clinical screenings. The mean number of Decayed, Missing and Filled Teeth (DMFT) was 6.4 ± 4.4. Sixty-four percent had moderate gingivitis and 73% had fair oral hygiene. ‘Recent visit to the dentist’ was associated with mother’s level of education (X^2^ = 22.06, p < 0.001) and father’s employment (X^2^ = 24.02, p < 0.001). The final regression model showed that grade (β = 0.31, p < 0.001), gender (β = 0.06, p < 0.03), recent visit to the dentist (β = −0.06, p < 0.03) and drinking fresh juices (β = −0.05, p < 0.05) were statistically significant in explaining the high level of caries in this sample.

**Conclusions:**

This study indicates that Palestinian adolescents in marginalised governmental schools suffer the highest burden of dental disease and are disproportionally impacted when compared to other same-age students in the region. A high burden of disease was directly associated with unfavourable dietary habits, poor oral hygiene practices and challenges to accessing dental care services, and was indirectly associated with father’s employment and mother’s level of education.

Dental diseases are the most prevalent chronic diseases worldwide: an estimated 5 billion people globally suffer from dental caries. Worldwide, 60%–90% of schoolchildren have dental cavities.^19^ Neglected dental diseases have very serious consequences such as unremitting pain, sepsis, reduced quality of life, lost school days, disruption to family life, and decreased work productivity.^18^ Oral health inequalities show that oral diseases disproportionally affected the vulnerable, marginalised and underserved populations. Studies show that these communities suffer the most disease and often have the least access to care.^5,18^

There is an emerging consensus that the success of one sector depends on the success of all other sectors. Both the general health and oral health of whole populations are largely determined by political, economic, environmental and social factors, the social determinants of health. Caries is a noncommunicable disease (NCDs) and shares a set of common risk factors with other NCDs, namely sugar, tobacco, alcohol and poor diet.^30^ Health conditions such as asthma, diabetes, obesity and caries are a few examples of conditions impacted by dietary habits and physical activities.^5^ Published literature on factors contributing to dental disease among adolescents indicate relationships between caries experience, children’s demographic and socio-economic indicators, and access to dental care.^28^ Body Mass Index (BMI) and dietary habits were also identified as important factors influencing levels of caries in any community, especially among adolescents.^27^ These factors are heavily influenced by cultural and social factors, which make studying dietary habits in addition to other social factors an important area of research in order to understand the social determinants of health, and predictors of disease in a certain population.^27^

In addition, the WHO’s Commission on Social Determinants of Health^29^ indicates that political conflict can impede access to health facilities and is considered a hazard to health. Areas under conflict and political unrest suffer from challenging health-services delivery and provision of care, including dental care due to the sociopolitical and administrative context, and the restrictions in movement and transportation. A striking example of these conditions is found in area C8 in the Occupied Palestinian Territories (OPT). The Oslo Accords that aimed to achieve a resolution to the conflict in OPT for a transitional period divided the OPT into three zones (Fig 1): 1. area A, 3% of the land, where the Palestinian National Authority (PA) assumed control of all civilian administration, including health and security: 2. Area B, 27% of the land, where the PA has civilian authority, but shares security responsibility with Israel; and 3. Area C, where the PA has no control over the remaining 70% of the land.^21^

**Fig 1:**
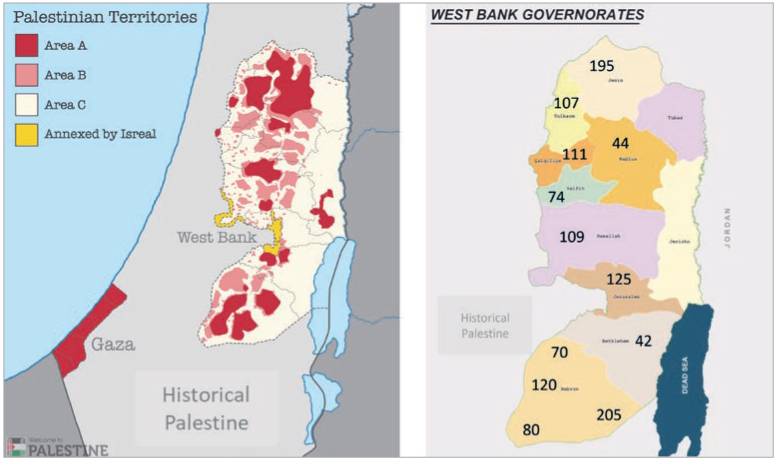
Distribution of areas A, B and C according to the Oslo Accord and demonstration of school districts, as well as number of students in each district.

Few studies in Palestine have assessed the dental caries experience among different populations. A convenience sample of 370 adults in the commercial centre of Palestine, Ramallah City, in 2015 showed that on average, an adult may have 9.5 Decayed, Missing and Filled Teeth (DMFT index for permanent teeth).^13^ In Jerusalem Governorate, in 2016, a study of a random sample of 152 pregnant women who visited prenatal health care centres demonstrated a DMFT score of 15.5.^14^ Other studies assessed the Early Childhood Caries (ECC) experience in younger children in different areas of Palestine. In Jenin, an urban centre in the north of the West Bank area, 76% of 1376 children aged 4-5 years had already experienced caries, with an average dmft (decayed, missing and filled teeth for primary teeth) of 2.46.^2^ In Nablus, another city between the middle and the north of the West Bank area, 79.2% of the 450 children aged 4–5 years had experienced caries, with an average of 4.5 dmft.^25^

The World Health Organization (WHO) Basic Oral Health Survey (BOHS) recommends screening children in 6th and 9th grades (ages 12 and 15 years, respectively) for caries and gingival health for global comparisons.^20^ Two studies in the northern cities of the West Bank area of the Palestinian Territories that screened 12-year-old children demonstrated that 84% and 54.4% of 12-year-old children had caries, with DMFT scores of 3.45^23^ and 5.4, respecitvely.^16^ ^A third study in Jerusalem involving 12-year-old students found DMFT scores to reach 4 in high caries-risk schoolchildren.^^24^

A more recent study by Abuhaloub et al,^1^ which reviewed the trends of caries and poor gingival health for a national sample of children 6, 12 and 16 years of age, showed less disease experience, especially for 12-year-old schoolchildren. Data in the previous study were extracted from the 1998-2013 Palestinian Ministry of Health School Dental Health Program (SDHP) annual reports. Results of the Abuhaloub et al^1^ study demonstrated lower DMFT scores than those that were reported in other studies for similar age groups.^16,23^ In the West Bank area, Abuhaloub et al^1^ reported a prevalence of 47% for caries among 12-year-olds and 49% among 16-year-olds. DMFT scores were 1.6 and 2.3 for 6th and 9th graders, respectively.

Another study,^6^ which targeted Palestinian refugees served by the United Nation Relief and Work Agency for Palestine refugees in the Near East (UNRWA) in 3 neighbouring countries in addition to the West Bank and Gaza, showed that caries experience among 12-year-old schoolchildren in refugee camps located in the West Bank was 85%. However, this study was limited to students attending UNERWA-operated schools which are mainly located in refugee camps and serve documented Palestinian refugees. Unlike schools operated under the UNERWA, governmental schools in Palestine which operate under the supervision of the Palestinian Authority do not have any oral health school programmes that offer preventive or therapeutic services.

In summary, published data on caries experience among Palestinian adolescents suggest a high burden that increases with age. However, few previous studies have investigated and identified factors that are unique to the culture, political conditions and society of the Palestinian population, and which may influence caries prevalence.

Therefore, this study assessed the prevalence of caries, plaque accumulation and gingivitis among a national sample of Palestinian adolescents attending marginalised schools in the West Bank area and investigated factors related to those conditions.

To the best of the authors’ knowledge, there has been no study of this kind conducted in the West Bank area of Palestine. It is important to establish a baseline in this marginalised group who are living in precarious and fragile circumstances. Focused attention on marginalised schools and hard-to-reach population groups can guide policy makers to tailor special interventions that meet their unique needs. This study will guide and inform interventions at a health-systems level as well as support integrating oral health in health and development programmes.

## Materials and Methods

### Sampling Technique

The School Support Program (SSP), launched in 2013, is a United States Agency for International Development (USAID)-funded initiative implemented by the American-Mideast Educational and Training Services, Inc (AMIDEAST)/Palestine, in partnership with the Save the Children organisation.^26^ SSP in collaboration with the Ministry of Education at the Palestinian Government (Palestinian Authority) analysed more than 1700 schools in the West Bank area of the Occupied Palestinian Territories (OPT) in order to identify the 50 most educationally marginalised in terms of poverty, educational achievement, drop-out rates, and underqualified teachers.^26^

**Fig 2:**
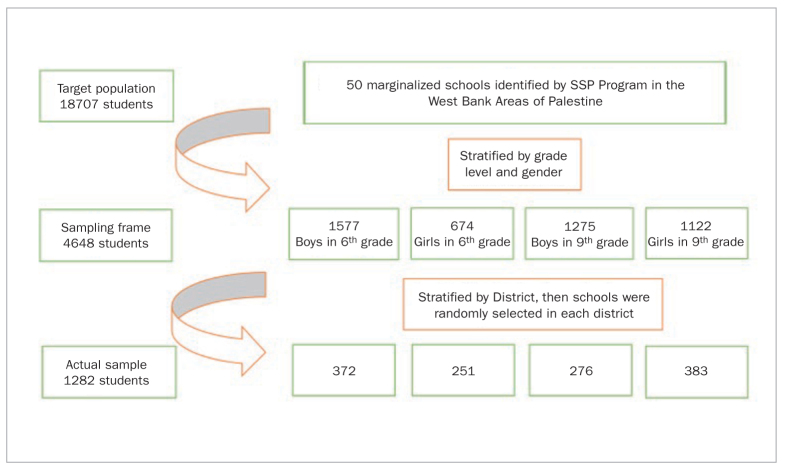
Flow chart of the stratified cluster sampling technique used in this study

Most of the schools included in SPP programme are located in Area C, as defined in the Oslo Accord.^8^ A map of the districts included in the sample and the Areas A, B and C are shown in Fig 1. The current study used a cluster-stratified sampling technique to select schools to participate in the study. Following the WHO recommendations on preferred ages for oral health screening, 20 students aged 12 and 15 years old in the 6th and 9th grades at the 50 SPP marginalised schools comprised our target population (n = 4688). Sample size calculation was carried out on our target population using a 95% confidence level and 3% margin of error. A minimum sample of 870 students was needed to ensure the accuracy of our results. Additionally, to increase the generalisability of our results, the fifty SPP marginalised schools were stratified by district (n = 12) and gender to select a representative proportionate random sample of 20 schools that included 1480 6th and 9th graders. All students in 6th and 9th grades in the sampled schools were recruited, and consent forms were sent to their parents to approve students’ participation. Only healthy students with no systemic conditions were included in the final analysis. A flow chart detailing the sampling technique is shown in Fig 2.

### Oral Hygiene Practices and Dietary Habits Survey Instrument

Students in the 6th and 9th grades were interviewed by senior dental students using a structured survey covering specific demographic data as well as their oral hygiene and dietary habits between September 2016 and May 2018. Demographic data captured information about mothers’ education level and fathers’ employment status. Oral hygiene habits were measured by asking about brushing and flossing frequency (never, sometimes, at least once a day). Access to dental care was quantified by the question, ‘When was your last visit to the dentist? In the past 6 months; in the past year; more than a year ago; never.’

Questions asked about diet were: number of meals a day (one; two; three), snacking habits (yes; no), frequency of added sugar (number of spoonfuls/day), milk consumption (cups/day), sweets and chocolate (times/day), fruits and vegetables (frequency/week), nuts and legumes (frequency/week), non-vegetable food such as eggs, fish, meat and poultry (frequency/week), junk food such as hamburgers, street food and fried chicken (frequency/week), carbonated drinks (frequency/week), sweetened juices (frequency/week), energy drinks (frequency/week), and fresh fruit juices (frequency/week). The questionnaire was tested for content validity by six experts in the field of public health and nutrition and for face validity by pilot testing the survey in 30 students of the same ages in our targeted sample.

### Oral Health Screening

Students’ weight, height, gingival health and caries experience were assessed by senior dental students. Senior dental students were trained and standardised on the WHO BOHS oral health screening criteria in three 2-hour sessions to carry out interviews and screening.^20^ Senior students at the time of examination would have passed three courses in paediatric dentistry, four courses in operative dentistry and two courses in community and preventive dentistry, which qualified them to examine children under supervision. A dental public health specialist, EK, confirmed all dental examinations before recording in files.^14^ The DMFT index was used to quantify the caries experience among adolescents. The same index for primary teeth (dmft) was used in combination with DMFT in mixed dentition, mainly for 6th graders. Examinations were carried out using disposable kits that included a mirror, an explorer with two ends, one sharp and the other blunt, a tweezer and an apron. Caries was assessed visually and gentle probing was used when in doubt about the presence of cavitation. Sterile gauze for drying and a hand flashlight for lighting were used for better visibility.

According to the WHO BOHS,^20^ DMFT scores for 12-year-old children are categorised as follows: very low: 0.1–1.1; low: 1.2–2.6; moderate: 2.7–4.4; high: 4.5–6.5; very high: > 6.5. The Gingival Index (GI)^15^ was used to describe whether the children had gingivitis. Averages of GI for each child were categorised as follows: 0 = healthy gingiva; 1 = mild gingivitis; 2 = moderate gingivitis; 3 = severe gingivitis. The Silness-Löe Plaque Index (PI)^15^ was used to assess plaque accumulation on children’s teeth, which reflects oral hygiene. PI was categorised as follows: PI 0: no observable plaque; PI 1: a thin film of plaque detected at the gingival margin by running a probe or explorer across the tooth surfaces; PI 2: a moderate amount of plaque detected along the gingival margin, plaque clinically visible; P3: heavy plaque accumulation detected at the gingival margin and in the interdental spaces. This index was measured at 4 points on 6 teeth, then averaged as follows: < 1: excellent oral hygiene; 1–1.9: good oral hygiene: 2–2.9: fair oral hygiene; ≥3: poor oral hygiene.^15^ Plaque and gingival assessment were carried out first using the blunt end of the explorer, then the children were instructed to brush their teeth using the provided toothpaste and toothbrush. Caries was assessed afterward.

### Statistical Analysis

All data were recorded using paper sheets and then transferred to an SPSS sheet. The proportion of children with DMFT > 0 was calculated to assess the prevalence of caries in this sample. To quantify the severity of oral conditions according to the WHO cut-off points, proportions of students who fell into each caries-experience and gingivitis category were generated. In addition, bivariate analysis attempted to associate demographic factors and dietary habits with their caries experience using Pearson’s correlation coefficient, t-test and ANOVA. Finally, a multi-variable model using linear regression suggested the important factors that influenced the high burden of disease in this sample. The outcome variable was the DMFT score. All analyses were carried out using SPSS v 20 (IBM; Armonk, NY, USA), with the statistical significance level set to 0.05.

Parental informed consents were collected by school administrative staff before the school visit. Ethical approval for all aspects of the study was obtained from Al-Quds University Scientific Research Ethics Committee. Administrative approval to conduct the study in the sampled schools was obtained from the Palestinian Government Ministry of Education.

## Results

One thousand two-hundred eighty-two (1282) students completed the in-person interviews and clinical screening, with a response rate of 86.6%. All parents of students in the designated samples consented to their children’s participation. Only students who were absent on the day of the visit were not included in the screening activity. Fifty percent of the sample were males and 51.4% were 9th-graders. Fifty-one percent of the students had fathers who were unemployed or who had non-regular jobs, 46% of the sample had mothers with less than a high-school education and 34% had mothers with only a high-school diploma.

**Fig 3:**
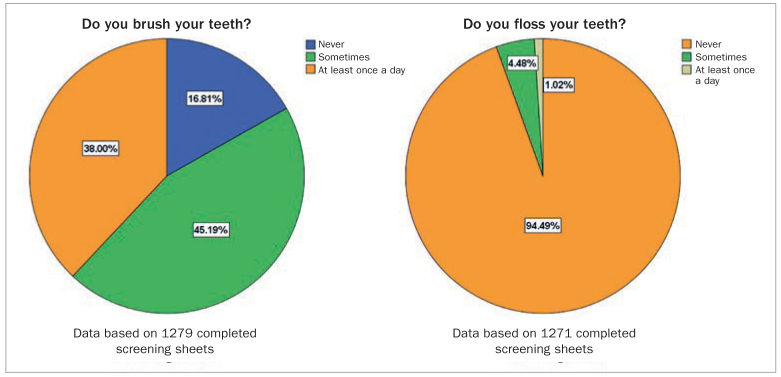
Toothbrushing and dental flossing practices in the study sample, n = 1282.

**Fig 4:**
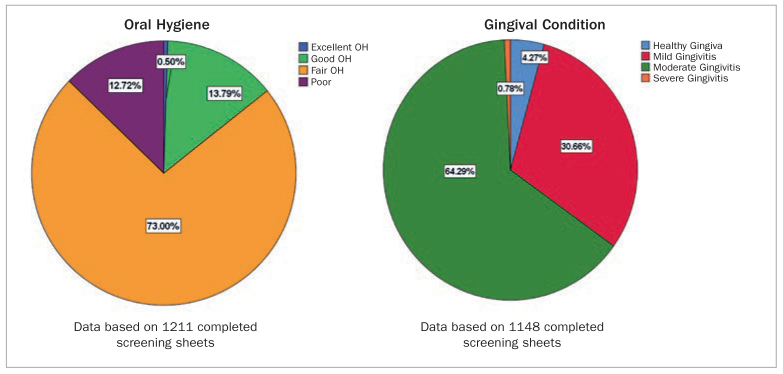
Oral hygiene index and gingivitis prevalence in the study sample, n = 1282.

Sixty-two percent of the children in the sample ‘never’ or ‘sometimes’ brushed their teeth and 94.5% never flossed between their teeth. Therefore, only 13.8% had good oral hygiene according to the PI and 64.3% had moderate gingivitis. Data about oral hygiene practices are shown in Fig 3, and the results of the Oral Hygiene Index and gingivitis prevalence in this sample are shown in Fig 4.

Regarding dietary habits, 69% had three meals a day and 86% snacked between meals. Twenty percent of 9th graders consumed energy drinks at least once a week compared to 11.8% of 6th graders (p < 0.001). More information about dietary habits can be found in Table 3.

**Table 1:** Caries experience (DMFT and DMFS) for 6th- and 9th-grade Palestinian students attending marginalised schools

	6th Grade (n = 623)	9th Grade (n = 659)	p-value	Male (n = 648)	Female (n = 634)	p-value
DMFT mean ± SD	4.9 ± 3.4	7.8 ± 4.7	<0.001	4.9 ± 4.3	6.9 ± 4.4	<0.001
DT mean ± SD	4.6 ± 3.2	70.0 ± 4.6	<0.001	5.5 ± 4.1	6.2 ± 4.1	<0.001
FT mean ± SD	0.1 ± 0.5	0.5 ± 1.24	<0.001	0.2 ± 0.8	0.4 ± 1.1	<0.001
DMFS mean ± SD	7.3 ± 60.0	11.2 ± 8.3	< 0.001	8.7 ± 7.6	9.9 ± 7.5	<0.001
DS mean ± SD	6.4 ± 4.9	9.3 ± 7.1	< 0.001	7.6 ± 6.5	8.2 ± 6.1	0.08
FS mean ± SD	0.2 ± 0.9	0.8 ± 20.0	< 0.001	0.4 ± 1.3	0.7 ± 1.9	< 0.001

**Table 2:** Caries experience of this sample categorised according to the WHO severity cut-off points

DMFT categories	6th Grade n (%)	9th Grade n (%)	p-value	Male n (%)	Female n (%)	p-value
Very low (0.1–1.1)	97 (16%)	65 (10%)	0.02	95 (15%)	67 (10%)	0.03
Low (1.2–2.6)	48 (8%)	28 (4%)	0.01	46 (7%)	30 (5%)	0.07
Moderate (2.7–4.4)	169 (27%)	81 (12%)	<0.001	142 (22%)	108 (17%)	0.08
High (4.5–6.5)	139 (22%)	109 (17%)	0.01	123 (19%)	125 (20%)	0.73
Very high > 6.5	170 (27%)	375 (57%)	<0.001	242 (37%)	303 (48%)	<0.001
	623 (100%)	658 (100%)		648 (100%)	633 (100%)	

**Table 3:** Dietary habits among schoolchildren in this sample

Dietary Habits	6th Grade Mean ± SD	9th Grade Mean ± SD	p-value
Sugar (spoons/day)	2.3 ± 1.8	2.7 ± 3.3	0.002
Milk (cups/week)	4.9 ± 6.3	2.8 ± 4.9	0.000
Carbonated drinks (times/week)	3.5 ± 3.6	3.8 ± 3.9	0.146
Sweetened juices (times/week)	3.8 ± 3.9	3.5 ± 3.1	0.207
Energy drinks (times/week)	0.3 ± 1.3	0.6 ± 1.8	0.001
Fresh fruit juices (times/week)	2.2 ± 2.5	20.0 ± 3.5	0.248

Thirty-four percent of our sample fell in the ‘overweight-obese’ or ‘obese’ category according to the most recent BMI cut-off points for children. 69% were classified as ‘normal weight’ and 6% as ‘underweight’.^4^

The prevalence of caries experience in this sample was 92%, with a prevalence of 93.5% in 15-year-old students (9th grade) and 90.4% in 12-year-old students (6th grade). The average DMFT score was 6.4 ± 4.4 for the whole sample, which places Palestinian students in this sample in the ‘high’ caries experience category according to the WHO classifications. The average of untreated decay measured by the Decayed Teeth (DT) component was 5.9 for the whole sample. This indicates that caries experience among this sample is mainly due to untreated caries, compared to an average of 0.3 for restored teeth, which constitutes the Filled Teeth (FT) component. Table 1 shows the DMFT, DT, MT and FT for 6th and 9th graders in this sample. Table 2 shows the caries experience in this sample categorised according to the WHO severity cut-off points.

Access to dental care was quantified by the ‘recent visit to the dentist’ variable. Data from this study showed that 23.4% never visited a dentist and 24.5% had visited a dentist more than a year ago. Meanwhile, 35.3% had had a visit during the past 6 months, but the main reason for the visit was pain relief.

Bivariate analysis demonstrated that plaque accumulation and gingivitis were positively correlated with caries experience, Spearman’s correlation ρ = 0.22 (p < 0.001) and ρ = 0.20 (p < 0.001), respectively. As expected, plaque accumulation and gingivitis were lower among students who brushed their teeth once or more a day (F = 23.7, p < 0.001), (F = 5.49, p < 0.001) and flossed their teeth once or more a day (F = 3.7, p < 0.02) and (F = 8.65, p < 0.001). Mother’s level of education correlated positively with the frequency of toothbrushing and flossing (ρ = 0.06 [p < 0.03] and ρ = 0.07 [p < 0.01], respectively).

In addition, bivariate analysis showed that 9th graders had higher DMFT scores than 6th graders (t = 3.77, p < 0.001) and female students suffered more disease than male students (t = 2.95, p < 0.001). Students who had mothers with higher levels of education had lower DMFT scores (ρ = -0.06, p < 0.03). Untreated dental decay also was also associated with mothers’ levels of education, where mothers with less than a high-school diploma had children with higher DT score than mothers who finished a college degree (F = 3.6, p < 0.03) with a post-hoc Tukey’s result of p < 0.02.

Moreover, the more recent the visit to the dentist, the higher DMFT score the student had (ρ = -0.06, p < 0.02). Recent visit to the dentist – an indicator of access to dental care – was associated with mother’s level of education (X^2^ = 22.06, p < 0.001), where higher levels of education were correlated with more ‘visit during the past 6 months’ answers and fewer ‘never been to the dentist’. In addition, father’s employment was associated with ‘last visit to the dentist’ (X^2^ = 24.02, p < 0.001). Adolescents whose fathers had regular jobs had a larger number of recent visits to dentists and fewer ‘never been to the dentist’ answers.

BMI was correlated with caries experience (ρ = 0.92, p < 0.001). Favourable dietary habits such as drinking milk and fresh juices were correlated with less disease (ρ = -0.08, p < 0.001 and ρ = -0.07, p < 0.01, respectively). Higher educational levels among mothers correlated with less carbonated-drink consumption among children, Spearman’s ρ = -0.08, p < 0.001.

In the final regression model, students’ grade (β = 0.31, p < 0.001), gender (β = 0.06, p < 0.03), recent visit to the dentist (β = -0.06, p < 0.03) and drinking fresh juices (β = –0.05, p < 0.05) were statistically significant and partially explained the high level of caries in this sample (r^2^ = 34 for the whole model).

## Discussion

This study focused on marginalised schools in the West Bank area. The results provide a first picture of the scale of the caries problem in this group and the scope of issues that must be considered in developing policy and programmatic interventions.

DMFT scores in the current study are higher than in other studies which investigated caries experience among Palestinian schoolchildren of the same age group. Our results were slightly higher than those reported in studies conducted in specific geographic areas in the OPT in governmental schools,^16,23^ but were significantly higher than in a study that reviewed the MOH SDHP results for the same age groups.^1^

In the study that reviewed the MOH SDHP caries experience scores,^1^ results of caries prevalence among children 12 and 16 years old showed a statistically significantly lower burden of disease than in the current study. In the previous report,^1^ 47% caries prevalence was found among 12-year-olds compared to 90.4% in our study and 49% among 16-year-olds in the previous study compared to 93% in the current study. DMFT scores were also higher in our study, 4.9 and 7.8 compared to 1.6 and 2.3 for 6th- and 9th-graders, respectively.

This can be partially explained by the types of schools selected. In our study, only governmental schools in that were categorised as marginalised by the SSP were included in the analysis, while in the SDHP review study,^1^ private and governmental schools from all the West Bank’s governorates participated in the screening; no political or economic criteria were applied.

Although some concerns were raised in the literature about the SDHP examiners’ training and calibration,^12^ these concerns were not validated further by other reports. The differences found between the DMFT scores in the current data and the national MOH reports seem to be mainly due to the sociopolitical context and the geographical location of the sampled schools.

When our results were compared to other countries in the region, we found that subjects in this study scored higher than subjects in similar studies in neighbouring countries using the same screening criteria. In Jordan,^22^ caries prevalence rates were 45.5% in 12-year-olds, and caries experience was 1.1 DMFT. In Syria,^3^ caries prevalence in schools in Damascus City was 79.1% and the mean DMFT was 2.03 ± 1.81. These marked differences can be explained by the target population in the current study from being marginalised schools rather than all governmental schools. However, scores from neighbouring countries are still lower than the SDHP scores, which suggests that Palestinian students suffer the highest burden of dental disease and are disproportionally affected compared to same-age students elsewhere in the region.

In addition to the uniqueness of the sample, which limited participants to students in marginalised schools, the current study investigated the prevalence of caries related to diet, BMI, oral hygiene practices and some demographic and social factors.

Social factors such as father’s employment and mother’s level of education were found to strongly determine caries experience in this group, both directly and indirectly. These factors influenced dietary habits, personal hygiene practices and, most importantly, access to dental care.

In addition to expected barriers to accessing dental care such as cost and time in general, in Area C, dental care utilisation is complicated by lack of dental health care providers, public or private, restriction of movements and transportation and the nonexistence of school oral-health programmes. This was demonstrated by the high need for treatment in this sample, which suffered from an average of untreated decay of DT of 5.9 compared to DT of 1.4 in the same age groups in non-marginalised schools.^1^

Beneficial dietary habits such as drinking milk and fresh juices instead of carbonated and sweetened juices were related to better oral health in this study. The shift from better to worse dietary habits affecting the two age groups in this study suggests that bad dietary habits increase between ages 12 and 15. Nutritional counselling should be extended into early childhood, and be integrated into school systems and curricula, with a focus on female students, who exhibited poorer dietary habits, higher BMI scores, and increased caries. Nutritional counselling would benefit oral health and general health in this group. A focus should be on milk consumption, which was much lower in the current sample than in global averages. Many countries and organisations recommend 3-4 cups of milk or other dairy intake daily;^10^ in the current study, students consumed on average 0.7 cups daily in the 6th grade and 0.4 cups daily in the 9th grade.

Energy drinks – which are significant sources of sugar, caffeine and acids – can cause obesity and caries. In the current sample, 20% of 9th-graders consumed energy drinks at least once a week. This is considered high compared to other global reports. In a sample of 31,070 adolescents across Europe, 68% reported drinking at least one energy drink in the previous year and 28% reported drinking one in the previous three days.^7^

Additionally, oral hygiene habits were clearly less than optimum in this study group. Irregular brushing and lack of knowledge about dental floss and its uses are evident in the high levels of plaque found and prevalence of moderate gingivitis as defined by the WHO criteria.^15,20^ Brushing with a fluoridated toothpaste may be the only method to deliver the protective benefits of fluoride to individuals who live in areas that lack added or natural fluoride. Supervised toothbrushing programmes in schools have been shown to be an effective population-based intervention.^9^

In general, raising awareness about increasing milk consumption, minimising consumption of carbonated and energy drinks, daily brushing and flossing, are three habits that need to be focused on in any educational campaign in this population to achieve better oral and general health. This agrees with the new definition of oral health adopted by the International Dental Federation (FDI) in 2017, which acknowledges the multifaceted nature and attributes of oral health. This definition aims mainly to emphasise the connection between oral health and general health, to urge health providers to better integrate oral health with general health, and to address the common factors that impact both.^11^ Thus, any health educational campaign needs to include oral health and vice versa.

Educational campaigns should be extended to the household and parents.^17^ The results suggest that mothers’ level of education may be a factor in disease levels, which reinforces the importance of educating mothers as well as children about good dietary habits and oral hygiene practices.

Sample size and technique suggest that the sample in this study is representative of students in targeted schools in marginalised areas in the West Bank area of OPT. However, the study still has some limitations, so that the study findings should interpreted with caution.

First, social desirability in the in-person interviews about dietary habits and hygiene practices may have limited the accuracy of data reported by the schoolchildren. Second, although having a dental public health specialist check students’ dental exams helped in standardising scoring the oral conditions, conducting inter-examiner reliability for students involved in the dental exams would have increased the reliability of the results in the current study. Third, the cross-sectional nature of the data collection limits the degree of accuracy of our assumptions about the factors that explained the disease burden. It will be interesting for future studies to longitudinally assess changes in diet and oral hygiene habits among adolescents and relate this to caries experience and gingival health.

## Conclusion

In this study, schoolchildren aged 12 (6th-graders) and 15 (9th-graders) years suffered from high DMFT scores that were mainly due to the untreated caries (DT). Dietary habits, oral hygiene practices, social factors and access to dental care are associated with oral disease in this sample, suggesting the need of interventions that target the problem of caries at multiple levels: personal, environmental and institutional.

Interventions at a personal level can be implemented to increase students’ awareness about oral health self-care skills in brushing and flossing. Health damaging behaviours are often adopted during adolescence, which have implications for non-communicable disease risk later in life. Therefore, health literacy programs including oral health should be incorporated into health-promoting schools as well as whole-school, whole community approaches. In addition, preventive measures such as supervised toothbrushing can be cost-effective interventions for this population.

At an environmental level, schools can enforce existing regulations to sell only healthy snacks at school cafeterias. This needs the support of the community through a school-community dialogue that addresses health comprehensively in this population and allows collaboration between different health promotion programmes.

Finally, at an institutional level, health authorities should improve access to dental care through establishing school health programmes, providing incentives to dentists to open practices in these areas and financing essential dental health services, preventive and therapeutic, through the public system as part of governmental attempts to implement universal health coverage.

Focusing on marginalised areas for oral health assessment can highlight and reflect the true burden of oral disease borne by these populations in health, social and economic terms. Policy-related interventions should be prioritised to implement changes in systems and address the institutional and environmental factors of populations most in need.

## References

[ref1] Abuhaloob L, Petersen PE (2018). Oral health status among children and adolescents in governmental and private schools of the Palestinian Territories. Int Dent J.

[ref2] Azizi Z (2014). The prevalence of dental carises in primary dentition in 4- to 5-year-old preschool children in Northern Palestine. Int J Dent.

[ref3] Ballouk M, Dashash M (2019). Caries prevalence and dental health of 8- to 12-year-old children in Damascus city in Syria during the Syrian Crisis; a cross-sectional epidemiological oral health survey. BMC Oral Health.

[ref4] Barlow SE (2007). Expert Committee. Expert committee recommendations regarding the prevention, assessment, and treatment of child and adolescent overweight and obesity: Summary report. Pediatrics.

[ref5] Benzian H, Monse B, Heinrich-Weltzien R, Hobdell M, Mulder J, van Palenstein Helderman W (2011). Untreated severe dental decay: a neglected determinant of low Body Mass Index in 12-year-old Filipino children. BMC Public Health.

[ref6] Biscaglia L, di Caccamo P, Terrenato I, Arrica MA, Seita A, Campus G (2019). Oral health status and caries trend among 12-year old Palestine refugee students: results from the UNRWA’s oral health surveys 2011 and 2016. BMC Oral Health.

[ref7] Committee on Toxicity of Chemicals In Food Consumer Products and the Environment Statement on the potential risks from ‘energy drinks’ in the diet of children and adolescents. https://cot.food.gov.uk/committee/committee-on-toxicity/cotstatements/cotstatementsyrs/cot-statements-2019/cot-statemet-on-energy-drinks.

[ref8] Declaration of Principles on Interim Self-Government Arrangements (Oslo Accords) United Nations Peacemaker, September 13th, 1993. https://peacemaker.un.org/israelopt-osloaccord93.

[ref9] Dickson-Swift V, Kenny A, Gussy M, de Silva AM, Farmer J, Bracksley-O’Grady S (2017). Supervised toothbrushing programs in primary schools and early childhood settings: A scoping review. Community Dent Health.

[ref10] Dror DK, Allen LH (2014). Dairy product intake in children and adolescents in developed countries: trends, nutritional contribution, and a review of association with health outcomes. Nutr Rev.

[ref11] Glick M, Williams DM, Kleinman DV, Vujicic M, Watt RG, Weyant RJ (2017). A new definition for oral health developed by the FDI World Dental Federation opens the door to a universal definition of oral health. J Public Health Dent.

[ref12] Kateeb E (2007). Evaluation of the Ministry of Health school oral health programme in the West Bank region of Palestine. East Mediterr Health J.

[ref13] Kateeb E, Sarhan M, Ghannam I (2015). Oral health status among convenient sample of Palestinian adults. Free Communication Sessions 21–40 and Poster Sessions 17–32. Int Dent J.

[ref14] Kateeb E, Momany E (2018). Dental caries experience and associated risk indicators among Palestinian pregnant women in the Jerusalem area: a cross-sectional study. BMC Oral Health.

[ref15] Löe H (1967). The Gingival Index, the Plaque Index and the Retention Index Systems. J Periodontol.

[ref16] Mahfouz M, Abu Esaid A (2014). Dental caries prevalence among 12–15 year old Palestinian children. Int Sch Res Notices.

[ref17] Nakre PD, Harikiran AG (2013). Effectiveness of oral health education programs: A systematic review. J Int Soc Prev Community Dent.

[ref18] Peres MA, Macpherson LMD, Weyant RJ, Daly B, Venturelli R, Mathur MR, Listl S (2019). Oral diseases: a global public health challenge. Lancet.

[ref19] Petersen PE, Bourgeois D, Ogawa H, Estupinan-Day S, Ndiaye C (2005). The global burden of oral diseases and risks to oral health. Bull World Health Organ.

[ref20] Petersen PE, Baez R J (2013). Oral health surveys: Basic Methods.

[ref21] Program of Assistance to the Palestinian People Palestinian economy. Country: land, people and government. https://unctad.org/en/PublicationsLibrary/tdb64d4_embargoed_en.pdf.

[ref22] Rajab L D, Petersen PE, Baqain Z, Bakaeen G (2014). Oral health status among 6- and 12-year-old Jordanian schoolchildren. Oral Health Prev Dent.

[ref23] Sabha B, Husseđn E, Abu Mowađs M, Hussein M, Muchađmer R (2010). The prevalence of dental caries in permanent dentition for 12-year-old school children in Northern Palestine. Süleyman Demirel Üniv Diş Hek Fak Derg Sayfa.

[ref24] Sagan-Cohen HD, Bajali M, Eskander L, Steinberg D, Zini A (2015). Dental caries status, socio-economic, behavioral and Biological Variables among 12-year-old Palestinian school children. J Clin Pediatr Dent.

[ref25] Samarah S (2015). Prevalence of early childhood caries and associated risk factors among preschool children in Nablus City/Palestine, Master’s thesis. http://scholar.najah.edu/sites/default/files/Suad%20Ayed%20Saed%20Samara.pdf.

[ref26] School Support Program, AMIDEAST, Palestine Success stories. 12 Years of reforming education in Palestine. October 1, 2018. https://www.amideast.org/news-resources/success-stories/12-years-of-reforming-education-in-palestine.

[ref27] Shivakumar S, Srivastava A, Shivakumar GC (2018). Body mass index and dental caries: a systematic review. Int J Clin Pediatr Dent.

[ref28] Tellez M, Zini A, Estupiñan-Day S (2014). Social determinants and oral health: an update. Curr Oral Health Rep.

[ref29] World Health Organization (2008). Social determinants of health in countries in conflict: a perspective from the Eastern Mediterranean Region.

[ref30] WHO oral health key facts (24 September 2018). World Oral Health Organization. https://www.who.int/news-room/fact-sheets/detail/oral-health.

